# Preliminary Evidence of Exogenous Hydrogen Peroxide Formation via Plant Transpiration: Toward a Nature-Based Solution for Air Quality and Climate Mitigation

**DOI:** 10.3390/bioengineering12111201

**Published:** 2025-11-03

**Authors:** Saman Samadi, Shabnam Sharifyazd, Ludwig Paul B. Cabling, Isaac Dekker, Barbara J. Hawkins, Heather L. Buckley, Kristian L. Dubrawski

**Affiliations:** 1 Department of Civil Engineering, University of Victoria, Victoria, BC V8W 2Y2, Canada; samansamadi@uvic.ca (S.S.); ssharifyazd@uvic.ca (S.S.);; 2 Department of Geography, University of Victoria, Victoria, BC V8W 2Y2, Canada; 3 Department of Biology, University of Victoria, Victoria, BC V8W 2Y2, Canada; 4 Department of Chemistry, University of Victoria, Victoria, BC V8W 2Y2, Canada; 5 Center of Advanced Materials and Related Technologies, University of Victoria, Victoria, BC V8W 2Y2, Canada; 6 Institute for Integrated Energy Systems, University of Victoria, Victoria, BC V8W 2Y2, Canada

**Keywords:** hydrogen peroxide, air quality, exogenous H_2_O_2_, bioremediation, plant biology, plant transpiration, atmospheric chemistry, nature-based solutions, climate change, microdroplets

## Abstract

Plants play critical roles as nature-based solutions to maintaining air quality and regulating biogeochemical cycles, yet the mechanisms underlying these complex systems remain poorly understood. Hydrogen peroxide (H_2_O_2_), a globally present atmospheric oxidant, shows well-documented diurnal variation, but no direct link to plant transpiration has previously been reported. This study aimed to determine whether plants can produce exogenous H_2_O_2_ through transpiration and condensation, thereby revealing a novel pathway by which plants influence proximal and potentially global atmospheric chemistry. To investigate this, we examined a natural plant system undergoing photosynthesis and transpiration; our work was inspired by recent laboratory findings where spontaneous H_2_O_2_ was generated during the condensation of water vapour into microdroplets in engineered systems. Condensed water collected near leaf surfaces revealed H_2_O_2_ concentrations of 1–5 ppm, verified using both commercial peroxide test strips and spectrophotometric titration. Importantly, H_2_O_2_ production occurred only under light conditions when plants were transpiring, while controls without plants or without light showed no detectable levels. A strong distance-dependence was also observed, with minimal to no H_2_O_2_ detected beyond 40 cm from leaves. These findings suggest that plant-driven formation of water vapour and subsequent condensation produces measurable H_2_O_2_, establishing a previously unrecognized mechanism with implications for air quality improvement, atmospheric oxidation processes, and climate change modelling and mitigation.

## 1. Introduction

Plants play a key role in air quality and biogeochemical cycle interactions [1,2] and are an important nature-based solution to mitigate the impact of human development on Earth. Plants transpire water (H_2_O) during photosynthesis; however, the fate of this transpired water vapour has been assumed to remain as H_2_O and follow classical atmospheric equilibrium chemistry and kinetics. Hydrogen peroxide, a powerful oxidant, exists in low concentrations in the atmosphere, although no linkage between terrestrial plants and atmospheric H_2_O_2_ has been reported [3,4]. H_2_O_2_ contributes to atmospheric cleansing as a reservoir for hydroxyl radicals [5], and as an oxidant of organic and biological contaminants. Endogenous H_2_O_2_ production by plants has been well-documented for signalling in cellular function, but exogenous H_2_O_2_ production by plants has not yet been demonstrated [6,7]. Importantly, endogenous concentrations within plant tissues are typically in the micromolar range, serving as signalling molecules [7], whereas our study shows exogenous release at significantly higher levels (ppm range), highlighting a distinct and previously overlooked pathway of oxidative output to the environment. In addition to regulating oxidant budgets, plants produce a rich suite of secondary metabolites (e.g., tannins, terpenoids, alkaloids, flavonoids) with broad antimicrobial activity that has been widely documented and leveraged in human applications [8]. Plants also deploy surface-secreted glycoproteins on aerial tissues—phylloplanins—that act at the first point of contact to deter pathogen establishment on leaves [9]. These plant-derived antimicrobial pathways are endogenous/biochemical and complement the exogenous, condensate-mediated H_2_O_2_ pathway explored here. Recently, it has been shown that H_2_O_2_ is spontaneously produced from water microdroplets (1–20 μm) without the addition of catalysts or additives, although the mechanistic explanation remains to be fully elucidated [10,11,12]. In vegetated ecosystems, the release of water as a byproduct of photosynthesis typically requires a vapour pressure deficit (VPD), where transpired water vapour typically mixes in the atmospheric column, contributing to local and global water cycles and climate dynamics. Whether plant transpiration and the recently discovered microdroplet condensation mechanism can contribute to local or atmospheric H_2_O_2_ concentrations has yet to be explored. Recent evaluations of nature-based solutions emphasize the role of plants in improving air quality and resilience against pollution, suggesting that oxidant pathways such as those mediated by H_2_O_2_ may be environmentally significant [13]. In this study, we report the first findings of H_2_O_2_ production via transpired water vapour across multiple plant species, suggesting that all plants are likely contributing to local, and perhaps global, atmospheric H_2_O_2_ concentrations, suggesting that H_2_O_2_-mediated oxidant pathways represent a potentially significant factor in ecosystem and atmospheric chemistry.

Implications of the potential of widespread plant-mediated H_2_O_2_ as a nature-based solution include local effects, such as impacting indoor air quality, and global effects, such as climate change mitigation. For indoor air quality, H_2_O_2_ is a strong oxidizing agent that denatures cellular components [14], suggesting that indoor plants could contribute to deactivation of pathogenic bacteria and viruses. In global biogeochemical cycles, H_2_O_2_ produced in vegetated areas may directly (via photochemical reactions), or indirectly (as a reservoir for HO_2_ radicals) contribute to the production of hydroxyl radicals (-OH*): the central oxidant of the lower atmosphere which controls the persistence of methane, carbon monoxide, nitrous oxide, and some ozone-depleting gases [5,15,16]. The broader climate relevance of these pathways is increasingly recognized, as recent reviews stress the need to integrate direct plant–atmosphere oxidative interactions into climate modelling frameworks [17]. While we demonstrate the first evidence for plant-mediated exogenous H_2_O_2_ production at the near-surface of plants, the implications and applications of these findings at scale require further study.

## 2. Materials and Methods

### 2.1. Experimental Set-Up

Our initial aim was to determine whether plants produce exogenous H_2_O_2_ via transpiration. We tested several plant species and examined the impacts of light intensity, humidity, distance from the leaf surface, and species on the resultant concentration of produced H_2_O_2_. We placed a plant, initially *Saintpaulia ionantha*, in a closed container. The soil surface of the pot was covered with foil, and the plant was placed inside a water and airtight polyethylene chamber. In experiments where peroxide test strips were used, strips were affixed to either the plant leaf surface, or to investigate spatial heterogeneity, strips were positioned at certain distances (0, 10, 40 cm) from the plant leaf surface. The chamber was then exposed to light at different intensities (300–600 µmol m^−2^ s^−1^) by a commercial LED plant growth bulb (5FR6 LED TUBE-24W). The light intensity was measured by an LCA-4 (ADC Ltd., Hoddesdon, Herts, UK)—a portable leaf chamber gas analyser. Relative humidity and temperature inside the chamber were monitored (Omega OM-62). The plant was left in the lit chamber for 1.5–8 h, after which the chamber was opened, and the strip or condensate was collected for H_2_O_2_ analysis. Controls included a chamber with no plant present; a chamber with a plant present but shielded from light; and a chamber with a plant and light present but leaves removed. All experiments were performed in duplicate. Radical and electron intermediates or droplet size distributions were not directly measured in this study. However, control experiments confirmed that both condensation and active transpiration are prerequisites for the detection of exogenous H_2_O_2_. In this study, the outcomes should be considered as initial findings that provide early evidence and open avenues for further comprehensive investigations.

### 2.2. Quantification of H_2_O_2_ Production

H_2_O_2_ was quantified in two ways. First, condensate was collected in the ranges of 100 to 300 µL in the chamber for analysis by chemical titration [18]. A 0.1 M potassium titanium oxalate (PTO, K_2_TiO(C_2_O_4_)_2_·H_2_O; ≥99.0%; Sigma-Aldrich, Saint Louis, MO, USA) solution was prepared before each batch of experiments. To develop a calibration curve, 300 µL of a hydrogen peroxide standard solution (H_2_O_2_, 30%; Fisher Scientific Co., Pittsburgh, PA, USA) with concentration between 0 and 20 ppm was added to 300 µL of PTO solution (Figure S1). Absorbance (400 nm) was measured by a SpectraMax M5 spectrophotometer (Molecular Devices, San Jose, CA, USA). A total of 300 µL of collected condensate was added to 300 µL PTO solution and [H_2_O_2_] was determined by the calibration curve (see Supporting Information for details). The average time from collection and measurement was approximately two minutes. In experiments where insufficient condensate was generated, or where we preferred not to open the chamber, peroxide test strips were used (0.5–25 ppm H_2_O_2_, Quantofix; Macherey-Nagel, Düren, Germany).

## 3. Results and Discussion

**Figure 1:**
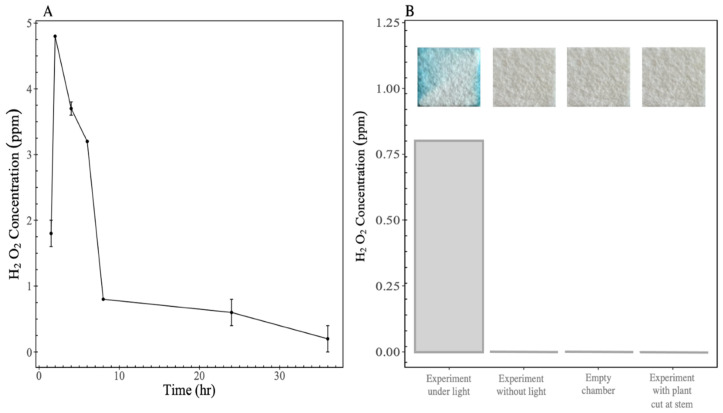
The effect of time and different conditions on hydrogen peroxide concentration. (**A**) Concentration of hydrogen peroxide produced by *Saintpaulia ionantha* under light at 600 µmol m^−2^ s^−1^ (n = 2). (**B**) Concentration of H_2_O_2_ produced at different conditions (from the left: exposed to light at 600 µmol m^−2^ s^−1^; no light control; empty chamber control, control with plant cut at stem). Data from chemical titration, photographs above data are peroxide test strips; blue color change indicates presence of H_2_O_2_.

In our first set of experiments in a controlled chamber (see Section 2), we observed H_2_O_2_ produced from transpiring *Saintpaulia ionantha* at concentrations of up to 5 ppm (~150 μM) after 2 h of transpiration (Figure 1A), decreasing to approximately 0 ppm after 36 h. No H_2_O_2_ was observed in controls with (i) no plant present, (ii) with a plant present without a light source, or (iii) with a plant present but cut at the stem after 12 h (Figure 1B). Further, no indication of any production of H_2_O_2_ over 12 h was observed in our controls (Figure 1B—inset photographs), as the peroxide test strips changed colour non-reversibly, i.e., no H_2_O_2_ was generated at any time in the controls. Our results suggest the observed H_2_O_2_ is generated only with plants undergoing photosynthesis and actively transpiring H_2_O. The transpired H_2_O vapour condensed either (i) within the control chamber, (ii) on the inside surface of the control chamber (where condensate was collected), or (iii) on the peroxide test strip itself, which explains the non-homogeneous colour patches detected on test strips. In any of these mechanisms, our results clearly indicate that condensation is a pre-requisite to the transformation of transpired H_2_O to detectable H_2_O_2_. Exogenous H_2_O_2_ was quantified in leaf-proximal condensate, with a maximum absolute amount of ≤18 μg detected per experiment. These findings represent an important first step, offering initial evidence that warrants broader investigations.

Water vapour is a byproduct of photosynthesis in plants; gas exchange via plant stomata takes in CO_2_ and releases H_2_O. At some distance from the leaf, depending on temperature, relative humidity (RH), and presence of seed nuclei, this water vapour will condense into a droplet. Recent research shows that condensing H_2_O droplets under certain conditions spontaneously form H_2_O_2_. Lee et al. [10] found that if the diameter of condensed H_2_O microdroplets is less than approximately 20 μm, high local electric field effects will cause autoionization of H_2_O, releasing a solvated electron and forming OH^−^ radicals which recombine to form H_2_O_2_. Other researchers propose a role of ultrasonic local humidification in the formation of H_2_O_2_ [11]. Regardless of mechanism, reports of H_2_O_2_ concentration and persistence are variable; Lee et al. [10] reported up to 30 μM (~1 ppm) by H_2_O nebulization; conversely, Lee et al. [12] generated H_2_O microdroplets by substrate cooling with H_2_O_2_ concentrations up to 4 ppm with persistence of only 5 min. In our work, we report H_2_O_2_ concentrations produced via plant transpiration of up to 6 ppm, with persistence of <1 ppm of several hours [19,20].

In our work, RH played a role in H_2_O_2_ concentration, possibly via controlling transpiration rate. Starting RH was ~37%, increasing to ~90% RH after 2 h, and remained at approximately that value for the duration of the experiments (Figure S5). As bulk RH rises, the vapour pressure deficit between bulk air and the boundary layer next to the stomata surface decreases, with a corresponding decrease in transpiration as per the Penman–Monteith equation [21].

We observed a sharp decrease in H_2_O_2_ concentration after 2 h (Figure 1A). It is likely that the generated H_2_O_2_ was reduced back to H_2_O via breaking of the weak peroxide bond, or via photochemical reactions [3,15]. Previous findings have shown a similar decrease in H_2_O_2_ concentration after microdroplet condensation on Peltier-cooled substrates, with a persistence of <5 min [12]. Alternatively, decreasing H_2_O_2_ could be a result of redox reactions with plant-mediated VOCs (not measured). Uncontrolled constituents in our experiments may also explain why H_2_O_2_ was persistent longer than observed in other experiments [12].

**Figure 2:**
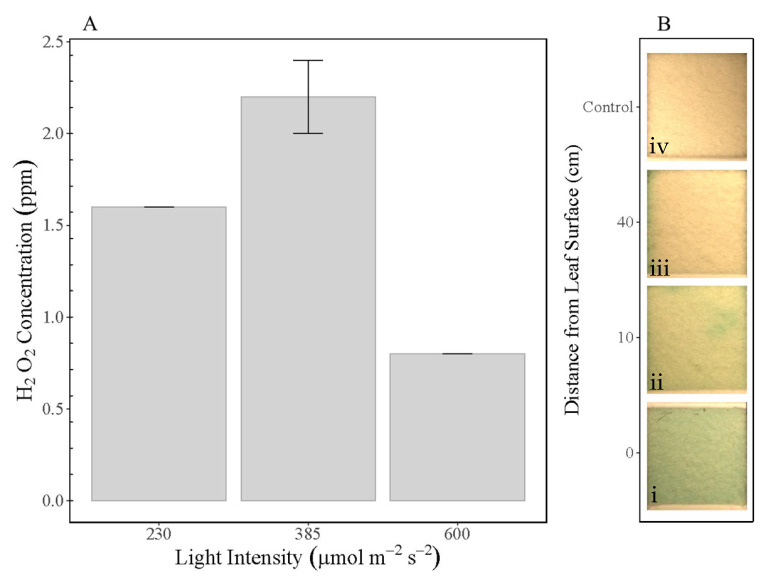
The effect of light intensity and distance on H_2_O_2_ concentration. (**A**) Relationship between light intensity and generation of H_2_O_2_ by *Saintpaulia ionantha* after 8 h (n = 2). (**B**) Photographs of peroxide test strips demonstrating the effect of distance from leaves after 8 h. Strips were positioned, from bottom: (**i**) attached to the leaves, (**ii**) 10 cm from the leaves, (**iii**) 40 cm from the leaves, (**iv**) control with no leaf. Blue color change indicates presence of H_2_O_2_.

We compared different light intensities to obtain spatial variation data at different distances from the leaf surface (Figure 2A,B). At three different light intensities (Figure 2A), we observe that an intermediate intensity (385 µmol m^−2^ s^−1^) results in higher measured H_2_O_2_ concentration (2.2 ± 0.2 ppm) than either low light intensity (230 µmol m^−2^ s^−1^, 1.6 ± 0.0 ppm), or high light intensity (600 µmol m^−2^ s^−1^, 0.8 ± 0.0 ppm) after 8 h. *Saintpaulia ionantha* may be light-saturated at an intermediate intensity [22], or the lower H_2_O_2_ concentration observed at a higher light intensity is a result of faster RH saturation, leading to a more rapid drop in H_2_O_2_ concentrations as described above.

Spatial heterogeneity in observed H_2_O_2_ was examined by placing peroxide test strips at various distances from the leaf surface (Figure 2B). We observed significantly higher H_2_O_2_ concentrations close to the leaf surface (0–10 cm) than further away (40 cm), the peroxide test strips closest to the leaf surface show more homogeneously saturated H_2_O_2_ detection, whereas at 40 cm away, little H_2_O_2_ is detected. Water vapour transpired from leaf surfaces will condense closer to the leaf surface due to the higher RH gradient, assuming the leaf continues to transpire water at relatively consistent rates, and turbulent air mixing is negligible in a closed system. As the water vapour begins to condense and coalesce, H_2_O_2_ concentrations may be dependent on the diameter of the resultant microdroplet [10], with little H_2_O_2_ formation at >10 μm. At distances far from leaf surfaces (40 cm), dilution of water vapour in air likely explains our lower observed H_2_O_2_ concentrations or alternatively, H_2_O_2_ may have already been formed and reduced back to H_2_O at greater distances from the leaf. Either way, these results further suggest that the plants are the source of the detected H_2_O_2_, as spatial homogeneity of H_2_O_2_ would have been likely had the source been from elsewhere (soil moisture, humidity in the chamber, etc.).

**Figure 3:**
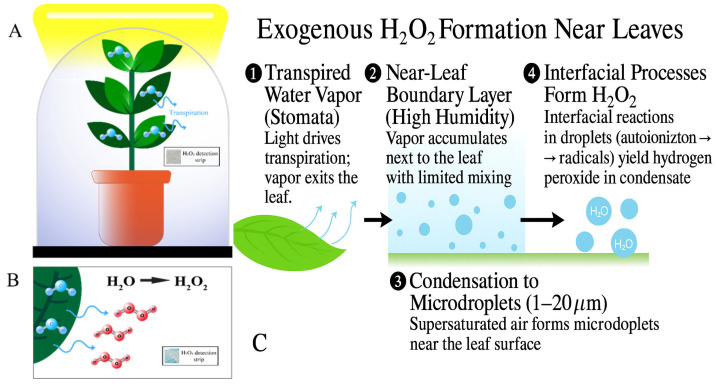
Experimental set-up scheme. (**A**) Schematic of experimental set-up for detecting plant-mediated H_2_O_2_. Commercial strips were located at 0, 10, and 40 cm from leaf surface. Condensate was collected for spectrophotometric titration. (**B**) Proposed mechanism of H_2_O_2_ formation away from leaf surface. Inset: photograph of peroxide detection strip after 2 h, strip at 10 cm from leaf surface. (**C**) Exogenous H_2_O_2_ formation near leaves, mechanism schematic.

Plants typically generate intracellular hydrogen peroxide as part of their metabolic signalling and stress–response networks, with concentrations generally maintained in the micromolar range by antioxidant enzymes [7,23]. The markedly higher values observed in our experiments indicate an external origin rather than leakage from cellular stores. This distinction underscores that the process we report is independent from classical endogenous pathways. In parallel, abiotic investigations have demonstrated that condensed microdroplets of water can yield H_2_O_2_ spontaneously due to interfacial physicochemical effects [10,11,12,19,20]. Our abiotic preliminary experiments confirmed these findings, although, unlike those engineered systems, our study suggests that transpiring plants naturally reproduce the microdroplet conditions necessary for such oxidant formation, thereby bridging atmospheric microdroplet chemistry with applied plant physiology. Moreover, plants are well-known to release a spectrum of atmospheric compounds, including volatile organic compounds (VOCs), tannins, polyphenols, and alkaloids [15,16,17,24]. These emissions contribute to oxidative processes and microbial regulation. The identification of exogenous H_2_O_2_ as an additional plant-derived flux broadens this framework, highlighting a new oxidative interface between vegetation and the surrounding environment. Figure 3C summarizes the hypothesized leaf–air pathway: transpired H_2_O accumulates in the near-leaf boundary layer, condenses to microdroplets, and interfacial processes yield H_2_O_2_; complementary atmospheric routes and sinks are noted.

We report consistent H_2_O_2_ production with different plants of the same species (Figure S2) and plants of different species (Figure S3), although different plants and different species produced different quantities of H_2_O_2_. While H_2_O_2_ production was consistent, variation among plants and species was likely due to differences in photosynthesis and transpiration rates from differing leaf surface area, stomata size, and morphology [25], leading to different H_2_O_2_ formation and persistence kinetics as previously described. A crassulacean acid metabolism (CAM) plant, *Aloe barbadensis* Miller, was also tested, showing minimal, but detectable H_2_O_2_ production in both night and day conditions (Figure S4). Trends and controls were similar in all plant species detected, suggesting all transpiring plants produce exogenous H_2_O_2_ to at least some degree.

The higher H_2_O_2_ concentrations in proximity to leaf surfaces have important implications in both air quality and biogeochemical cycles. For air quality, exogenous H_2_O_2_ production by plants may have implications in indoor air quality (e.g., hospitals); high-density regions (such as megacities) and rural regions may be impacted by forest fires, but likely only in close proximity to transpiring plants [26]. Our work further implicates plants as a viable nature-based solution for air quality improvement, including against pathogenic bacteria and viruses, and we are currently undertaking preliminary experiments. The findings of this study may also complement current research on the fate of plant-sourced biogenic volatile organic compounds, alkaloids, and polyphenols [27,28,29]. In biogeochemical cycles, H_2_O_2_ generation by plants may or may not impact boundary layer atmospheric H_2_O_2_ concentrations [30,31,32]. The established major mechanism for the formation of hydrogen peroxide in the troposphere is the bimolecular self-reaction of the hydroperoxyl radical (HO_2_) via photochemical chain reactions in the troposphere [33]. However, tropospheric H_2_O_2_ concentrations have been found to decrease with increasing latitude [34], increase with increasing vegetation density (i.e., inside a forest vs. the forest perimeter), [35] and vary by time of day and seasonality [15]. These imply greater H_2_O_2_ concentrations near vegetation with high net primary productivity (NPP), suggesting, at least in theory, a potential role of exogenous H_2_O_2_ production in atmospheric H_2_O_2_ concentrations at the landscape scale. However, the relative magnitude of our results on landscape and global processes remains to be explored. Further studies to elucidate the contribution to exogenous H_2_O_2_ production by plants require considering photochemical dynamics, including the role of seasonal and diurnal variation on the formation of hydroxyl radicals [36], the reaction of alkenes and ozone (O_3_) in the presence of water vapour [24], and the decomposition of the hydroperoxyl radical [33]. If the contribution of exogenous H_2_O_2_ production by plants is found to be significant at a global scale, transpiration may be an overlooked negative feedback process affecting methane concentration in the atmosphere and could potentially impact climate change models and forecasts [4,20,31,32]. Future mechanistic studies (e.g., catalase quenching, radical probes, O_3_/BVOC scrubbing, and droplet-size control on Peltier substrates) will be essential to connect these environmental implications with the underlying interfacial versus gas–phase pathways.

## 4. Conclusions

In this study, we provide the first experimental evidence that plants can produce exogenous hydrogen peroxide through transpiration under controlled conditions. Our results demonstrate that water vapour released during photosynthesis can, upon condensation near leaf surfaces, generate measurable concentrations of H_2_O_2_ without the need for external catalysts, additives, or engineered systems. This finding introduces a novel mechanism for plant–environment interaction, expanding the known roles of vegetation beyond carbon sequestration and VOC release to include potential oxidative contributions to atmospheric chemistry.

We observed that H_2_O_2_ generation is highly dependent on factors such as light intensity, distance from the leaf surface, and relative humidity, indicating that environmental microconditions play a critical role in the dynamics of this process. Importantly, this phenomenon was consistent across multiple plant species, suggesting that exogenous H_2_O_2_ production may be a widespread, yet previously overlooked, function of plant transpiration. The implications of this process are significant: from potential indoor air quality enhancement (via passive oxidative disinfection), to participation in local and regional oxidant cycles that influence the persistence of greenhouse gases such as methane and carbon monoxide.

Future research should explore this mechanism under semi-natural and outdoor conditions at the landscape scale, measure and model transpiration rates and variability in H_2_O_2_ production rates, further investigate the role of abiotic humidity and condensation on H_2_O_2_ production rates, and integrate these findings into atmospheric and climate models. Additionally, the interaction of plant-emitted H_2_O_2_ with other atmospheric constituents (e.g., VOCs, particulate matter) warrants further study. These efforts could help clarify the broader environmental relevance of plant-mediated H_2_O_2_ production and its potential application as a nature-based solution for environmental remediation and climate change mitigation. It should be emphasized that the present study reports preliminary findings, which highlight the novelty of exogenous H_2_O_2_ production by plants, while underscoring the need for further comprehensive studies under a wider range of conditions.

## Data Availability

The original contributions presented in the study are included in the article/Supplementary Materials, further inquiries can be directed to the corresponding author.

## Supplementary Materials

The following supporting information can be downloaded at https://www.mdpi.com/article/10.3390/bioengineering12111201/s1, Figure S1. Experimental readings of absorbance versus H_2_O_2_ concentration (data points connected by a line for visual clarity). Figure S2. Hydrogen peroxide concentration by different replicates of *Saintpaulia ionantha* plant under 600 µmol m^−2^ s^−1^ irradiance for 2 h (n = 2, error bars represent SD of H_2_O_2_ measurement). Error bars indicate standard deviation (SD) of duplicate measurements (n = 2). Figure S3. Hydrogen peroxide concentration produced by *Saintpaulia ionantha*, *Peperomia obtusifolia*, and *Epipremnum aureum* produced under 600 µmol m^−2^ s^−1^ irradiance after 4 h (n = 2, error bars represent SD of H_2_O_2_ measurement). Figure S4. Analysis of H_2_O_2_ production with representative crassulacean acid metabolism (CAM) plant, *Aloe barbadensis* Miller, during night and day without artificial light. Photographs of H_2_O_2_ strips directly after removing plant from control volume. A. Control (overnight, no plant), B. CAM plant overnight (10 p.m. to 8 a.m); C. CAM plant during daytime (8 a.m. to 1 p.m.). Figure S5. Relative humidity inside the chamber over 8 h experiment (*Saintpaulia ionantha,* 600 µmol m^−2^ s^−1^ irradiance).
