# Supplementary material for: Preliminary Evidence of Exogenous Hydrogen Peroxide Formation via Plant Transpiration: Toward a Nature-Based Solution for Air Quality and Climate Mitigation
Source: Bioengineering (Basel). 2025 Nov 3;12(11):1201. doi: 10.3390/bioengineering12111201 (PMC12650404; doi:10.3390/bioengineering12111201)
Supplement: Supplementary file 1 [file bioengineering-12-01201-s001.zip › bioengineering-3887775-supplementary.pdf]

Supporting Information

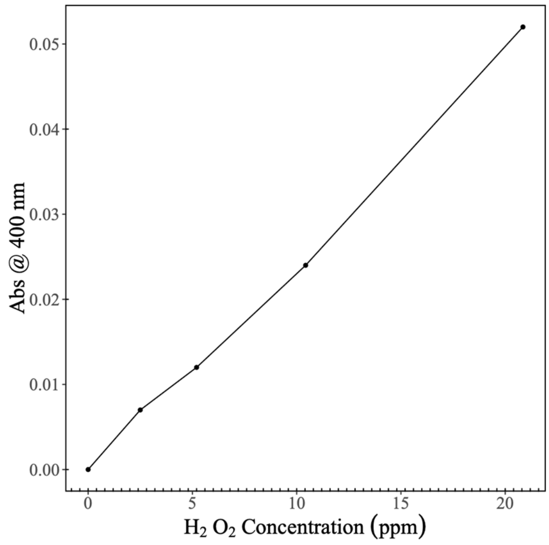

Figure S1. Experimental readings of absorbance versus H_2O_2 concentration (data points connected by a line for visual clarity).

Absorbance/H_2O_2 concentration calibration curve. Absorbance (400 nm) measured with SpectraMax M5 spectrophotometer (Molecular Devices).

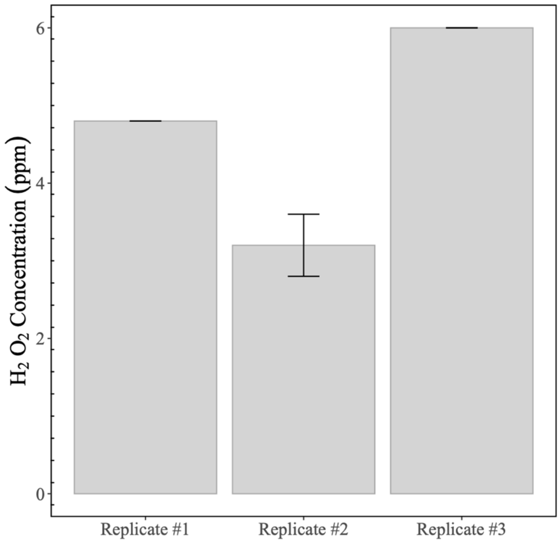

Figure S2. Hydrogen peroxide concentration by different replicates of *Saintpaulia ionantha* plant under 600 µmol m^{-2} s^{-1} irradiance for 2 h (n=2, error bars represent SD of H_2O_2 measurement). Error bars indicate standard deviation (SD) of duplicate measurements (n=2).

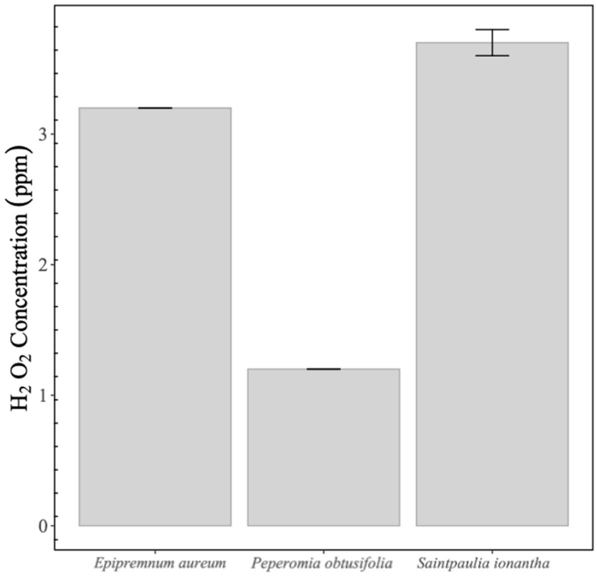

Figure S3. Hydrogen peroxide concentration produced by *Saintpaulia ionantha*, *Peperomia obtusifolia*, and *Epipremnum aureum* produced under 600 μmol m^{-2} s^{-1} irradiance after 4 h (n=2, error bars represent SD of H_2O_2 measurement).

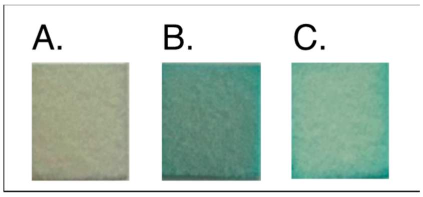

Figure S4. Analysis of H_2O_2 production with representative crassulacean acid metabolism (CAM) plant, *Aloe barbadensis* Miller, during night and day without artificial light. Photographs of H_2O_2 strips directly after removing plant from control volume. A. Control (overnight, no plant), B. CAM plant overnight (10pm to 8am); C. CAM plant during daytime (8am to 1pm).

- Panel A (Control, overnight, no plant): The pale colour indicates *no detectable* H_2O_2.
- Panel B (CAM plant overnight, 10 pm–8 am): The darker/greenish colour corresponds to a *detectable* H_2O_2 *level*, which is consistent with the physiology of CAM plants. Specifically, stomata in CAM species such as *Aloe barbadensis* open at night to take in CO_2, leading to transpiration during the night. This transpiration explains the observed exogenous H_2O_2 production at night.
- Panel C (CAM plant daytime, 8 am–1 pm): The intermediate colour indicates *slightly higher* H_2O_2 *levels than the control but lower than Panel B*. This is expected, since stomata in CAM plants remain mostly closed during the day to conserve water, leading to reduced transpiration and consequently reduced H_2O_2 release, even though photosynthesis continues internally with stored CO_2.

Importantly, all strips in this experiment were placed inside the chamber at distances of 0 cm (attached to the leaf), and the observed colour intensity corresponds qualitatively to relative H_2O_2 detection at that position.

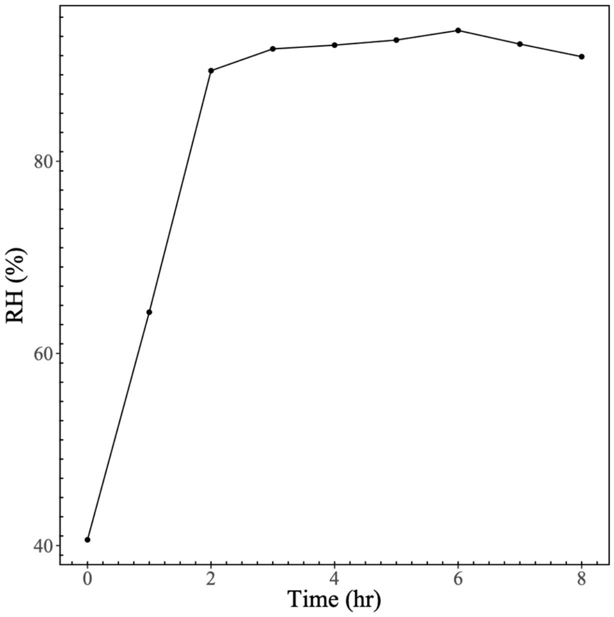

Figure S5. Relative humidity inside the chamber over 8 h experiment (*Saintpaulia ionantha,* 600 µmol m^{-2} s^{-1} irradiance).

Several control conditions were performed, including chambers with no plant, no irradiation, and no leaves. In all these controls, no significant increase in relative humidity (RH) was detected. Therefore, the observed rise in RH shown in Figure S5 can be directly attributed to plant transpiration.
